# Muscarinic Receptors Associated with Cancer

**DOI:** 10.3390/cancers14092322

**Published:** 2022-05-07

**Authors:** Gloria M. Calaf, Leodan A. Crispin, Juan P. Muñoz, Francisco Aguayo, Tammy C. Bleak

**Affiliations:** 1 Instituto de Alta Investigación, Universidad de Tarapacá, Arica 1000000, Chile; kcrispi@gestion.uta.cl (L.A.C.); jpmunozb@academicos.uta.cl (J.P.M.); tammy.bleak@biofiredx.com (T.C.B.); 2 Laboratorio de Oncovirología, Programa de Virología, Instituto de Ciencias Biomédicas (ICBM), Facultad de Medicina, Universidad de Chile, Santiago 8380000, Chile; faguayo@med.uchile.cl

**Keywords:** muscarinic receptors, breast, gastric, lung, colorectal, prostate, glioblastoma, liver, cancer

## Abstract

**Simple Summary:**

Recently, cancer research has described the presence of the cholinergic machinery, specifically muscarinic receptors, in a wide variety of cancers due to their activation and signaling pathways associated with tumor progression and metastasis, providing a wide overview of their contribution to different cancer formation and development for new antitumor targets. This review focused on determining the molecular signatures associated with muscarinic receptors in breast and other cancers and the need for pharmacological, molecular, biochemical, technological, and clinical approaches to improve new therapeutic targets.

**Abstract:**

Cancer has been considered the pathology of the century and factors such as the environment may play an important etiological role. The ability of muscarinic agonists to stimulate growth and muscarinic receptor antagonists to inhibit tumor growth has been demonstrated for breast, melanoma, lung, gastric, colon, pancreatic, ovarian, prostate, and brain cancer. This work aimed to study the correlation between epidermal growth factor receptors and cholinergic muscarinic receptors, the survival differences adjusted by the stage clinical factor, and the association between gene expression and immune infiltration level in breast, lung, stomach, colon, liver, prostate, and glioblastoma human cancers. Thus, targeting cholinergic muscarinic receptors appears to be an attractive therapeutic alternative due to the complex signaling pathways involved.

## 1. Introduction

In the last decade, with the development of successful treatments, the mortality index has increased in developed countries [1,2]. Among these chronic diseases, cancer has become common and is even being called by some authors the pathology of the century [3]. Cancer is one of the most important diseases of this decade, since around 9.6 million cancer-associated deaths occurred only in 2018 according to the WHO, and the cost associated with cancer has rapidly incremented over several years, reaching approximately USD 1.16 trillion in 2010 [4]. Thus, cancer represents a serious global health issue, even after years of scientific efforts and pharmacological improvements [5].

Carcinogenesis is a multistep process that involves molecular and cellular changes intimately related. These steps are identified as initiation, promotion, and progression [6]. Different causes have been identified as responsible for cancer initiation, such as smoking or even viruses [7], but the origin of this disease is due to multiple causes [1]. During cancer progression, cells create different features, thus exerting many responses to several treatments [8]. Therefore, the environment around the normal and cancer cells, the interchange of substances, and the particular phenotype to apply new cancer therapies should be considered [9]. Over the past years, different methods to treat cancer have been studied, starting from surgery, chemotherapy, radiation, and chemoradiation to other more sophisticated ones, such as immunotherapy, stem cell, or bone marrow transplant, that can be used alone or in combination depending on the type and characteristics of the tumor, therefore, allowing the rise of new therapeutic regimens [3]. Recent new knowledge in molecular biology, genetics, immunology, and technology has helped to develop new pharmacological approaches with more effective and fewer-side-effect therapies; hence, different therapeutic targets have been studied since the 1980s [3] and they have been approved for cancer treatment, such as hormone therapies, signal transduction inhibitors, gene expression modulators, inducers/inhibitors, and monoclonal antibodies [4]. The molecular mechanism of these new approaches allows them to selectively act on cancer cells, decreasing the side effects [10].

Gene expression analysis was carried out by TIMER2.0, a web source of information that systematically evaluates the clinical impact of different immune cells in various cancer types through three components: Immune Association, Cancer Exploration, and Immune Estimation, each with several modules to comprehensively investigate tumor immunological, clinical, and genomic features, such as the Gene module that provided the statistical analysis carried out by Spearman’s p test in the Immune Association component; the Gene_DE module that provided statistical analysis carried out by the Wilcoxon test; the Gene_Outcome module with the analysis carried out by Z-Score test, and the Gene_Corr module that provided the statistical analysis carried out by the Spearman’s test in the Cancer Exploration component. TIMER2.0 uses the following dataset: TCGA Breast Invasive Carcinoma (BRCA); lung adenocarcinoma (LUAD); lung squamous cell carcinoma (LUSC); stomach adenocarcinoma (STAD); colon adenocarcinoma (COAD); liver hepatocellular carcinoma (LIHC); prostate adenocarcinoma (PRAD); and glioblastoma multiforme (GBM) [11]. A *p* < 0.05 was considered to be significant.

The ability of muscarinic agonists to stimulate growth and muscarinic receptor antagonists to inhibit tumor growth has been demonstrated for breast, melanoma, lung, gastric, colon, pancreatic, ovarian, prostate, and brain cancer. Thus, this work aimed to study the correlation between epidermal growth factor receptors and cholinergic muscarinic receptors, the survival differences adjusted by the clinical stage, and the association between gene expression and immune infiltration level in breast, lung, stomach, colon, liver, prostate, and glioblastoma human cancers. Therefore, targeting cholinergic muscarinic receptors appears to be an attractive therapeutic alternative due to the complex signaling pathways involved.

## 2. Cancers Associated with Muscarinic Receptors

### 2.1. Breast Cancer

Breast tumors are classified into five molecular subtypes, such as luminal A (LumA), luminal B (LumB), HER2 overexpression (Her2), basal, and normal-like tumors, each one with a distinct clinical outcome [12,13]. The mammary epithelium comprises cells that express receptors to respond to ovarian hormones, including estrogen receptor alpha (ERα) [14]. ERα, a typical marker of the luminal epithelial phenotype in breast cancer cells, is a good indicator of breast cancers that responds to endocrine therapy. Estrogen (E2)/ERα signaling promotes the differentiation of mammary epithelia along a luminal/epithelial lineage through transcriptional activation of luminal/epithelial-related transcription factors [15]. Authors described that E2 promoted breast cancer proliferation, migration, and invasion [16]. However, others stated that only a low concentration of E2 promoted the proliferation of breast cancer cells through ERα, whereas a high concentration induced apoptosis independent of the presence of ERα [17]. Normal proliferating mammary epithelial cells rarely express Erα, whereas ERß has been detected in 30–47% of proliferating epithelial cells [18]. One aspect of breast cancer is that up to 75% expresses ER, being an indication of a certain level of dependency on estrogen for cell growth [19]. Furthermore, different gene expressions depend on the subtype of cancer, i.e., ER-positive and ER-negative, and there are common functions shared among the group of genes part of a specific subtype of cancer, for instance, common functions in cell death, regulation of cell proliferation, intracellular signaling cascades, response to oxygen and hormones, among others; thus, gene signatures are related to similar functions and pathways despite its individuality as a gene [20].

The usual therapy for ER-positive breast cancer is the endocrine therapy that blocks the growth-promoting effects of estrogen via ER, such as Tamoxifen (Z)-2-[4-(1,2-diphenylbut-1-enyl) phenoxy]-ethyldimethylamine citrate, which is a selective estrogen receptor modulator (SERM) and is the most successful treatment for early and advanced stages of ER-positive breast cancers. Tamoxifen has been used as an adjuvant in patients under surgery or radiation; however, many patients with this breast cancer usually show de novo or acquired resistance requiring very aggressive treatment afterward, such as chemotherapy [19]. In a more clinical context, these markers (ER presence) have not only shown the usefulness of the therapy in positive cases (60 to 80%), but they have also indicated the aggressiveness of hormone-receptor-negative cases, which calls for chemotherapeutic agents [21].

Epidermal growth factor receptor (EGFR) is strongly linked to cancer progression and it is a promising therapeutic target for breast cancer [22]. Most breast tumors with cutaneous metastases are human epidermal growth factor receptor 2 (HER2)-positive subtype; anti-EGFR molecules, such as Trastuzumab and pertuzumab, have exhibited disappointing results, due to the lack of specificity and frequent adverse side effects; however, Pyrotinib (approved only in China), a new small-molecule tyrosine kinase inhibitor that irreversibly blocks EGFR and HER2, as well as human epidermal growth factor receptor 4 (HER4), may have a dual therapeutic impact against HER2 and mucin 1, improving the outcome of patients [23,24].

Inhibitors of small molecules appeared to target elements as part of important signal pathways, such as MAPK, PI3K/AKT/mTOR, BCR-ABL, and EGFR [25,26]; thus, Gefitinib or Erlotinib are selective EGFR inhibitors and Imatinib inhibitors of Bcr-Abl tyrosine kinase [27]. EGFR family comprises four types of tyrosine kinase receptors, including ErbB1 (HER1), ErbB2 (HER2), ErbB3 (HER3), and ErbB4 (HER4), and it is highly expressed in cancer cells [28,29]. EGFR overexpression activates pathways related to proliferation, angiogenesis, cell motility, metastasis, and other tumor-related processes [30,31], and the EGFR pathways associated with cell growth are RAS–RAF–MEK–ERK MAPK and AKT–PI3K–mTOR [29,32]. Several data have demonstrated the relationship between G-protein-coupled receptors (GPCRs) and EGFR via transactivation in different cancers [17,32,33].

Acetylcholine (ACh) is recognized as the principal neurotransmitter in the central and peripheral nervous systems, key in processes such as learning memory, and autonomic and muscular contraction [33], where nicotinic and muscarinic receptors are necessary to maintain communication between cells and organ homeostasis [34,35]. However, its role is not limited to the nervous and neuro-muscular systems; thus, considering its presence in cancer cells has led different investigators to study its influence on the progression of different cancers [36,37]. Since ACh is also related to tumor progression, questions have emerged about mAChRs and their presence in non-neuronal systems, and their role in processes such as survival, differentiation, and proliferation [38].

The muscarinic acetylcholine receptors (mAChRs), which belong to the G-protein-coupled receptor family, regulate a wide type of biological processes [39,40] by the activation of the EGFR pathway [41,42]. Five receptor subtypes named M1–M5 have been identified [43], which are encoded by the cholinergic receptor muscarinic 1–5 (*CHRM1*–*5*) genes [44]. Since some cancers overexpress certain receptors and its mediation is normally associated with other effectors, such as EGFR, the sole study of mAChRs is not enough to establish a possible treatment or a unique novel therapeutic target; for instance, with nicotinic receptors, data are confirming the activation of the PI3K/AKT/MAPK pathway after nicotinic receptor stimulation; therefore, a synergetic effect after stimulating both receptors should be important to approach and to consider [45,46]. The ability of muscarinic agonists to stimulate growth and the M3 receptor antagonists to inhibit tumor growth has been demonstrated for breast, melanoma, lung, gastric, colon, pancreatic, ovarian, prostate, and brain cancer [16,17,18,47,48].

Authors reported that mAChRs were overexpressed in tumor cell lines compared with normal breast cell lines, for instance, in the MCF-7 cell line, an estrogen-dependent cell line derived from breast carcinoma [18,49,50]. High levels of M2 and M3 receptors were present in MCF-7, and activation of these muscarinic receptors stimulated the cell growth in the same cell line [49,50]; a higher expression of M3 and M4 was reported to promote cell proliferation, whereas MCF-10A cell line lacked mAChRs [18,49]. Similarly, cells derived from the normal murine mammary gland (spontaneously aroused from BALB/c mice) did not exhibit mAChR expression, while a metastatic cell line (LMM3R) derived from those cells dramatically overexpressed it (40-fold) [51,52]. Although mAChRs were expressed in some tumor cells, the M3 receptor appeared to be involved in tumor progression [53]; for example, muscarinic activation induced cell proliferation, and, when there was a blocking of the M3 receptor, such proliferation was inhibited in vivo and in vitro [36,54], and modulation of the cell growth by receptor agonists and antagonists was observed [55].

Other studies showed that autoantibodies in the sera of patients were detected in the breast [56,57,58], and a characteristic of immunoglobulin G (IgG) contributed to tumor development in the early stages of breast cancer by the activation of mAChRs [43]. Lombardi et al. (2013) observed that IgG had an important role in the tumor vascularization fundamental in tumor growth and metastasis process in the MCF-7 tumor cell line; furthermore, IgG from patients in stage I contributed to the VEGF-A expression and production, and upregulation of matrix metalloproteinase-9 (MMP-9) by the activation of mAChRs (M3/M4); these effects were diminished by a muscarinic antagonist as atropine and increased by a cholinergic agonist as carbachol [57].

#### 2.1.1. Correlation between EGFR Expression and Cholinergic Muscarinic Receptors in Breast Invasive Carcinoma

**Figure 1:**
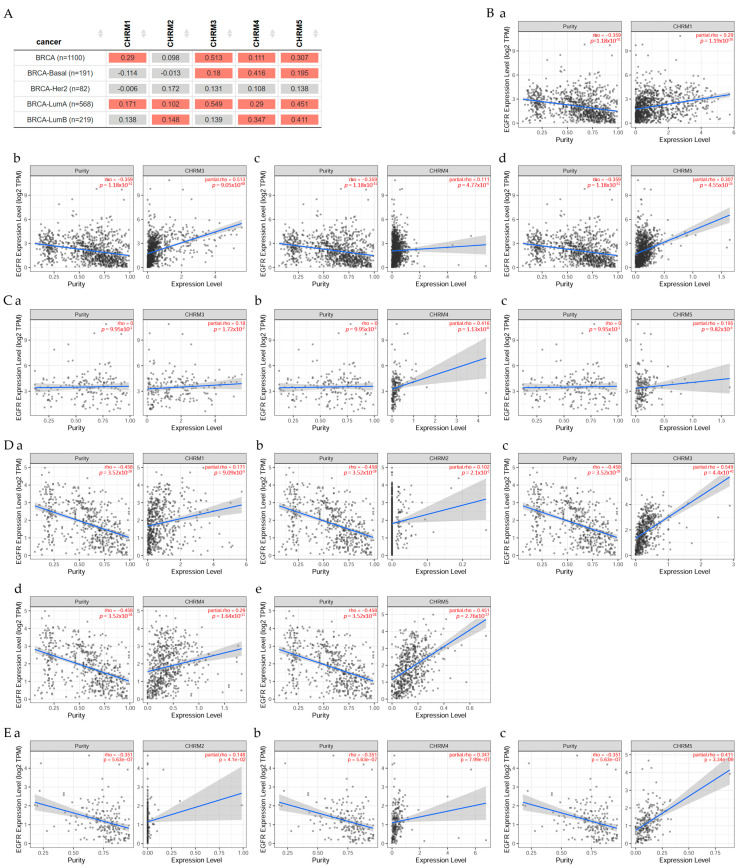
The co-expression pattern of genes across various cancer types was evaluated with the Timer2.0 Exploration component. (**A**) The heatmap table, from the Gene Corr Module, shows the correlation between *EGFR* expression and cholinergic muscarinic receptor 1–5 (*CHRM1–5*) in all breast invasive carcinoma (BRCA), BRCA-Basal, BRCA-Her2, BRCA-LumA, and BRCA-LumB with purity adjustment. The red color indicates a statistically significant positive correlation (Spearman’s, *p* < 0.05), and gray denotes a non-significant result. Scatter plots show significant correlations between *EGFR* expression and (**B**) (**a**) *CHRM1*, (**b**) *CHRM3*, (**c**) *CHRM4*, and (**d**) *CHRM5* in all BRCA (*n* = 1100); (**C**) (**a**) *CHRM3*, (**b**) *CHRM4*, and (**c**) *CHRM5* in BRCA-Basal (*n* = 191); (**D**) (**a**) *CHRM1*, (**b**) *CHRM2*, (**c**) *CHRM3*, (**d**) *CHRM4*, and (**e**) *CHRM5* in BRCA-LumA, and (**E**) (**a**) *CHRM2*, (**b**) *CHRM4*, and (**c**) *CHRM5* in BRCA-LumB with tumor purity (left panel) and with the expression level estimated by TIMER2.0 (right panel) [11].

Results in Figure 1 indicated there was a significant difference (*p* < 0.05) between *EGFR* and *CHRM1* in all BRCA, but no significant difference in BRCA-Basal and BRCA-Her2 patients. *CHRM2* was not significant in all BRCA nor BRCA-Basal or BRCA-Her2; however, there was a significant difference (*p* < 0.05) between *EGFR* expression and *CHRM3*, 4, and 5 in all BRCA and BRCA-Basal patients. There was a significant difference (*p* < 0.05) between *EGFR* and *CHRM1*, *CHRM2*, *CHRM3*, *CHRM4*, and *CHRM5* in BRCA-LumA; and between *EGFR* and *CHRM2*, *4*, and *5* in BRCA-LumB patients.

#### 2.1.2. Survival Difference in Breast Invasive Carcinoma Adjusted by the Stage Clinical Factor

**Figure 2:**
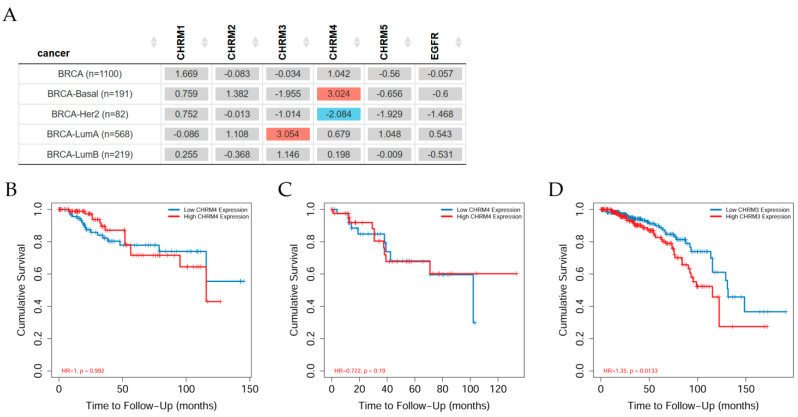
Associations between gene expression and tumor features in TCGA were evaluated with the exploration component of the Timer2.0 web resource. (**A**) The heatmap table generated by the Gene Outcome Module shows the survival analysis of cholinergic receptor muscarinic 1–5 (*CHRM1*–*5*) and *EGFR* expression in breast invasive carcinoma (all BRCA), BRCA-Basal, BRCA-Her2, BRCA-LumA, and BRCA-LumB adjusted by the clinical-stage factor. The red color indicates a statistically significant increased risk (Z-score, *p* < 0.05), the blue color indicates a significantly decreased risk (Z-score, *p* < 0.05), and gray denotes a nonsignificant result. The Kaplan–Meier (KM) curves provide detailed information about the corresponding significant relationship between gene expression and survival of (**B**) *CHRM4* in BRCA-Basal (*n* = 191), (**C**) *CHRM4* in BRCA-Her2 (*n* = 82), and (**D**) *CHRM3* in BRCA-LumA (*n* = 568) at low and high expression given by cumulative survival and time to follow-up estimated by TIMER2.0 [11].

Overall survival differences among patients stratified by mAChRs expression level in several types of cancer can be seen in Figure 2. Results indicated there was no significant difference between receptor gene expression and tumor features in breast invasive carcinomas (BRCA) adjusted by stage clinical factor. However, *CHRM3* expression showed a significant (*p* < 0.05) increased risk in BRCA-LumA; the Kaplan–Meier (KM) curves indicated that *CHRM3* showed a significant (*p* < 0.001) difference between low and high expression in stage 4 in BRCA-LumA (B); *CHRM4* had a significant (*p* < 0.05) increased risk in BRCA-Basal, but a significant (*p* < 0.05) decreased risk in BRCA-Her2 adjusted by stage clinical factor; KM plots showed that *CHRM4* expression had a significant (*p* < 0.01) difference between low and high expression in stage 4 in BRCA-Her2.

Abnormal activation of EGFR family kinases, including ErbB1 (HER1), ErbB2 (HER2), ErbB3 (HER3), and ErbB4 (HER4), results in excessive cell proliferation, angiogenesis, and apoptosis avoidance [59]. Following ligand attachment, HER2 is activated by dimerization, which commences intracellular auto-phosphorylation of tyrosine residues and initiates cell proliferation signaling pathways [60]. HER2 inhibition, which became a standard targeted therapy after HER2 overexpression was found in up to 25% of breast cancer patients, providing the best results for its treatment [61]. Muscarinic receptors are expressed in MDA-MB-231 tumor cells and cell viability is reduced when carbachol or arecaidine propargyl ester, a nonselective or selective mAChR M2 agonist, is combined with paclitaxel, resulting in downregulation of EGFR and other transporters; and those combined drugs inhibit tumor cell migration and have antiangiogenic effects in vitro and in vivo (in immune depressed mice), providing compelling evidence for using mAChR M2 as therapeutic targets for triple-negative cancers [62].

#### 2.1.3. Association between Gene Expression and Immune Infiltrates in Breast Invasive Carcinoma

**Figure 3:**
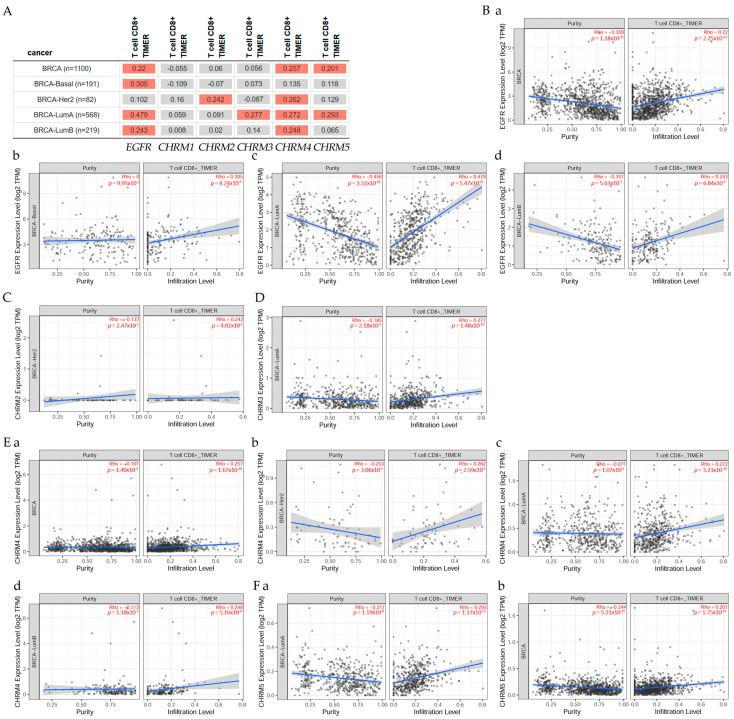
Association between immune infiltrates and gene expression evaluated with TIMER2.0 web resource. (**A**) The heatmap table, an output from the Gene Module, shows the association between *EFGR*, cholinergic muscarinic receptor expression 1–5 (*CHRM1*–*5*), and immune infiltration level of T cell CD8^+^ across breast invasive carcinomas (BRCA). The red color indicates a statistically significant positive correlation (Spearman’s, *p* < 0.05) and gray denotes a nonsignificant result. Scatter plots show the correlation between (**B**) *EGFR* expression and T cell CD8^+^ in (**a**) all BRCA (*n* = 1100), (**b**) BRCA-Basal (*n* = 191), (**c**) BRCA-LumA (*n* = 568), and (**d**) BRCA-LumB (*n* = 219); (**C**) *CHRM2* and T cell CD8+ in BRCA-Her2 (*n* = 82); (**D**) *CHRM3* and T cell CD8^+^ in BRCA-LumA; (**E**) *CHRM4* and T cell CD8^+^ in (**a**) all BRCA, (**b**) BRCA-Her2, (**c**) BRCA-LumA, and (**d**) BRCA-LumB; and (**F**) *CHRM5* and T cell CD8^+^ in (**a**) all BRCA and (**b**) BRCA-LumA with tumor purity (left panel) and with the infiltration level of T cell CD8^+^ estimated by TIMER2.0 (right panel) [11].

The composition and abundance of immune cells in the tumor microenvironment have a significant impact on tumor progression and immunotherapy efficacy (Figure 3). However, due to the limitations of direct measurement methods, computational algorithms are commonly used to infer immune cell features from a large number of tumor transcriptome profiles. TIMER2.0 tools analyze the relationship between immune infiltrates and genetic or clinical features through four components that explore cancer-related associations in The Cancer Genome Atlas (TCGA) cohorts [11].

This work studied the correlation between *EGFR* and *CHRM1–5* and it was found that all muscarinic receptors were present in BRCA-LumA patients; however, only *CHRM3*, *4*, and *5* in BRCA-Basal and *CHRM2*, *4*, and *5* in BRCA-LumB, indicating different muscarinic therapeutical solutions for these subtypes of breast cancer. Survival difference showed that *CHRM3* expression indicated an increased risk in BRCA-LumA, whereas *CHRM4* expression showed an increased risk in BRCA-Basal, but there was a decreased risk in BRCA-Her2 adjusted by the clinical factor.

Results indicated that there was a significant difference between muscarinic receptors and breast cancer subtypes when the clinical stage was analyzed. It was observed that *CHRM1−5* and *EGFR* were significantly (*p* < 0.001) higher in stages 3 and 4 than in other stages in all BRCA. It was also observed that *CHRM1*–*5* and *EGFR* were significantly (*p* < 0.001) higher in clinical stage 4 than in other stages in BRCA-LumA. Then, *CHRM1−5* and *EGFR* were significant (*p* < 0.01 and *p* < 0.05, respectively) in stage 4 in BRCA-LumB. Results also showed that *CHRM1*, *3*, *4*, and *5* were significant (*p* < 0.01), *CHRM2* was significant (*p* < 0.001), and *EGFR* was also significant (*p* < 0.05) in stage 4 in BRCA-Her2; however, *CHRM1*–*5* and *EGFR* showed no significant difference in any of the clinical stages in BRCA-Basal.

Therefore, targeting cholinergic muscarinic receptors appears to be an attractive therapeutic approach to subtypes of breast cancer. It is important to highlight that this approach does not help to solve the problem with BRCA-Basal patients, since there was no significant difference in any of the stages and across the various muscarinic receptors.

### 2.2. Lung Cancer

Lung cancer is still one of the leading cancer-related deaths worldwide [63] with about 9.6 million deaths only in 2018 according to the WHO [64]. There are two classifications of lung cancer: small cell lung carcinoma (SCLC) and non-small cell lung carcinoma (NSCLC), with 20% and 85% of cases worldwide, respectively [34,65]. Within NSCLC are large cell carcinoma, lung adenocarcinoma, squamous cell carcinoma, and neuroendocrine lung carcinoid tumor [66,67]. Among the risk factors for lung cancer are lifestyle, such as cigarette smoking [68]; about 55 substances have been identified in cigarettes considered as carcinogenic by the IARC, causing DNA adducts and DNA methylation [68,69]. Other risk factors are environmental, such as particulate matter [70,71,72,73]; diet/nutrition [74,75]; and previous pulmonary conditions, such as chronic bronchitis, tuberculosis, or pneumonia history [76,77]. Epidemiological studies have stated there is a direct relation between cigarette smoking and developing lung cancer [78], and this is connected with cancer incidence and mortality [68].

Song et al. (2003) reported the existence of both nicotinic and muscarinic receptors in small cell lung carcinoma [79]. ACh and cholinergic-related components were also demonstrated in human lung cancers, inducing adhesion migration and invasion [34] in a sort of autocrine manner via a cholinergic autocrine loop [65], while the M3 autocrine loop was highly expressed in SCLC; this expression was detected after the examination of 24 SCLC samples in which 17 were positive, and where choline acetyltransferase (ChAT) was also expressed [80]. In addition, 60% of all the squamous cell carcinoma of the lung (SCC-L) tumors expressed ChAT, suggesting a role of the cholinergic system in the progression of lung cancers [81]. Overexpression of M3 was observed in NSCLC patients with poor survival rates [82]. However, it was reported that ACh also served as a paracrine and autocrine growth factor for bronchial epithelial cells, SCLCs, SCC-Ls, and lung adenocarcinomas [83,84,85,86]. Crosstalk between the M3 receptor and EGFR was suggested in lung cancer, enhancing processes such as proliferation, migration, and invasion due to ACh activity [34].

Data suggested that lung cancer had different systems to increase the production of ACh, such as upregulation of ChAT and VAChT, or downregulation of acetylcholinesterase (AChE) [34,65,87,88,89]. A previous study on the effects of different muscarinic agonists on lung cancer proposed the activation of the EGFR/PI3K/AKT pathway due to M3 activation with partial participation of matrix metallopeptidases (MMPs), but the full mechanism is still unclear [32]. However, it was reported that arecoline, an alkaloid with muscarinic and nicotinic effects, induced migration and activation of EGFR, with further cascade activation involving c-Src and FAK signaling in the A549 lung cancer cell line [90]. The mechanism of action proposed considered the transactivation of EGFR through M3/MMP7-cleaved EGF-like ligand, with the following c-Src/FAK signaling pathway activation [17,32,91,92]. Such effect was then revoked by 4-DAMP and matrilysin, a muscarinic antagonist and a neutralizing antibody of matrix metalloproteinase, respectively [90].

Other drugs, such as Genitinib, a selected inhibitor of EGFR tyrosine kinase, and Dasatinib, a kinase inhibitor (including the c-Src family), reversed the effects of arecoline [90], indicating a relationship between muscarinic effects and EGFR effectors. Results showed that carbachol induced EMT with further activation of the ERK signaling pathway via M1 and M3 [91]. Yang et al. (2016) observed that ACh interacted not only with the mAChR, but also with EGFR, activating ERK and AKT, being a communication link between these two pathways to potentiate cell proliferation [92]. Other studies confirmed this communication, specifically the ability of M3 in EGFR transactivation [41,93]. In NSCLC, upon M3-mediated EGFR transactivation, the signaling pathway was activated, thus phosphorylating ERK1/2 and AKT [32,94,95], and, under a muscarinic antagonist (R2-8018), the PI3K/AKT and MEK/ERK1/2 pathways were inhibited, therefore, inhibiting tumor growth [82]. Other studies using M3 antagonists demonstrated that small cell and non-small cell human lung cancer decreased their proliferative features and cell growth in vitro [96] and in vivo [80].

#### 2.2.1. Correlation between EGFR Expression and Genes Associated with Cholinergic Muscarinic Receptors in Lung Adenocarcinoma and Lung Squamous Cell Carcinoma

**Figure 4:**
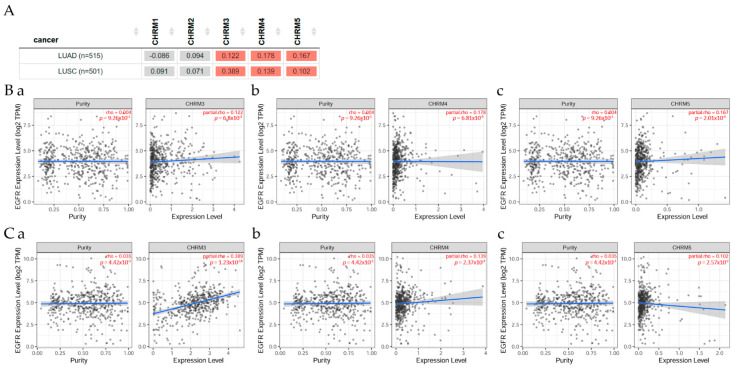
Co-expression pattern of genes across various cancer types explored with TIMER2.0. (**A**) The heatmap table, an output from the Gene Corr Module, shows the correlation between *EGFR* expression and cholinergic receptor muscarinic 1–5 (*CHRM1–5*) in lung adenocarcinoma (LUAD) and lung squamous cell carcinoma (LUSC) with purity adjustment. The red color indicates a statistically significant positive correlation (Spearman’s, *p* < 0.05) and gray denotes a non-significant result. Scatter plots show the significant correlations between *EGFR* expression and (**B**) (**a**) *CHRM3*, (**b**) *CHRM4*, and (**c**) *CHRM5* in LUAD (*n* = 515); and (**C**) (**a**) *CHRM3*, (**b**) *CHRM4*, and (**c**) *CHRM5* in LUSC (*n* = 501) with tumor purity (left panel) and with the expression level estimated by TIMER2.0 (right panel) [11].

Figure 4 presents the correlation between *EGFR* expression and genes associated with mAChRs in lung adenocarcinoma (LUAD) and lung squamous cell carcinoma (LUSC) patients, evaluated with TIMER2.0 analysis tools [11]. There was a positive correlation (*p* < 0.05) between *EGFR* and *CHRM3*, *CHRM4*, and *MCHR5* expression in LUAD and LUSC patients, respectively.

#### 2.2.2. Survival Difference in Lung Adenocarcinoma and Lung Squamous Cell Carcinoma Adjusted by the Stage Clinical Factor

**Figure 5:**
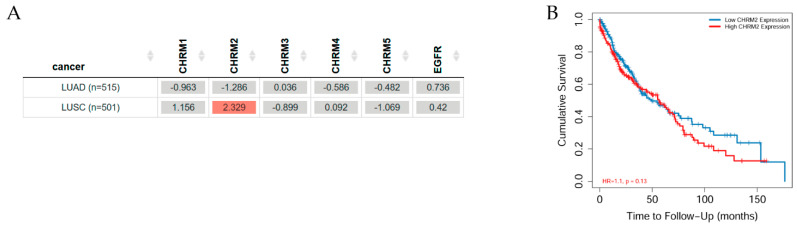
Associations between gene expression and tumor features in TCGA were evaluated with the Timer2.0 Exploration component. (**A**) The heatmap table generated by the Gene Outcome Module shows the survival analysis of cholinergic receptor muscarinic 1–5 (*CHRM1*–*5*) and *EGFR* expression in lung adenocarcinoma (LUAD) and lung squamous cell carcinoma (LUSC) adjusted by the clinical-stage factor. The red color indicates a statistically significant increased risk (Z-score, *p* < 0.05) and gray denotes a nonsignificant result. The Kaplan–Meier (KM) curves provide detailed information about the corresponding significant relationship between gene expression and survival of (**B**) *CHRM2* in LUSC (*n* = 501) at low and high expression given by cumulative survival and time to follow-up [11].

Overall survival differences among patients stratified by mAChRs expression level in lung cancer can be seen in Figure 5. There was mostly no significant difference between receptor gene expression and tumor features in lung (LUAD and LUSC) cancer adjusted for the patient stage; however, *CHRM2* expression showed a significant (*p*< 0.05) increased risk in LUSC; the Kaplan–Meier (KM) curves indicated that *CHRM2* showed a significant (*p* < 0.01) difference between low and high expression in stage 3 and 4 in LUSC (B).

When comparing LUAD through the different disease stages for all *CHRM1*–*5*, it was observed that there was a significant (*p* < 0.001) difference for stages 2, 3, and 4. However, in LUSC, there was a significant difference from stages 3 to 4. There was a significant difference for stage 3 (*p* < 0.05) and stage 4 (*p* < 0.01) in *CHRM1*, *3*, and *5*; furthermore, there was a significant (*p* < 0.01) difference for stage 2 for *CHRM2*, and there was a significant (*p* < 0.05) difference for *CHRM4*, as well as for *EGFR.*

#### 2.2.3. Association between Gene Expression and Immune Infiltration Level in Lung Adenocarcinoma and Lung Squamous Cell Carcinoma

**Figure 6:**
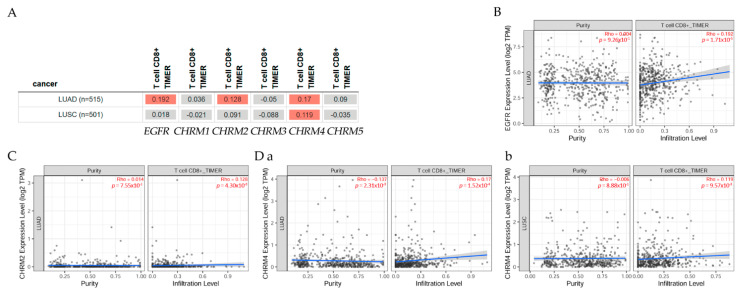
TIMER2.0 Immune component was used to evaluate associations between immune infiltrates and gene expression. (**A**) The heatmap table, an output of the Gene Module, shows the association between *EFGR*, cholinergic muscarinic receptor expression 1-3 (*CHRM1-3*), and immune infiltration level of T cell CD8^+^ across lung adenocarcinoma (LUAD) and lung squamous cell carcinoma (LUSC). The red color indicates a statistically significant positive correlation (Spearman’s, *p* < 0.05) and gray denotes a nonsignificant result. Scatter plots show the correlation between (**B**) *EGFR* expression and T cell CD8^+^ in LUAD (*n* = 515); (**C**) *CHRM2* expression and T cell CD8^+^ in LUAD; and (**D**) *CHRM4* expression and T cell CD8^+^ in (**a**) LUAD and (**b**) LUSC (*n* = 501) with tumor purity (left panel) and with the infiltration level of T cell CD8^+^ estimated by TIMER2.0 (right panel) [11].

Correlation of gene expression with immune infiltration levels in diverse cancer types can be seen in Figure 6. The gene module according to data on TIMER2.0 web analysis tools [11], using Spearman’s *p* values, allowed us to select genes and visualize the correlation of its expression with immune infiltration level in (A) *EGFR* and diverse muscarinic receptor types. (B) Results indicated there was a positive significant (*p* < 0.05) correlation between *EGFR* gene expression and immune infiltrates, considering the T cell CD8^+^ by TIMER in (B) LUAD, but it was not significant between *EGFR* and T cell CD8^+^ in LUSC. (C) There was a positive significant (*p* < 0.05) correlation between *CHRM2* gene expression and immune infiltrates, considering the T cell CD8^+^ by TIMER in LUAD, but a not significant (*p* < 0.05) correlation in LUSC. (D) Results indicated there was a positive significant (*p* < 0.05) correlation between *CHRM4* gene expression and immune infiltrates, considering the T cell CD8^+^ by TIMER in (a) LUAD and (b) LUSC.

### 2.3. Stomach or Gastric Cancer

Stomach cancer, also known as gastric cancer (GC), is one of the most common low-survival-rate cancers in the world [97], accounting for 783,000 deaths worldwide in 2018 [64]. According to the Lauren classification, gastric cancers can be divided into intestinal and diffuse cancers, depending on their microscopic and macroscopic features [94]. Among the risk factors are environmental and genetic alterations [95]. Hence, genetic mutations, family history, and previous gastric surgery remain unchangeable risk factors [98,99]. It has been observed that there is a high risk in those who smoke tobacco [100], and another reported risk factor is H. pylori, which, in 1994, was declared a carcinogen (class I) by the International Agency for Research on Cancer, demonstrating an evident correlation with GC [101]. Gastric cancer has more prevalence in males than females, and it is correlated to the grade of development of countries, being more common in developed countries [97]. However, maintaining proper weight and healthy dietary habits, such as being low in salt and high in fruits and vegetables, may avoid gastric-related problems [102,103].

In the stomach, ACh is secreted from vagus nerves and Dclk1+ tuft cells, and the gastric acid is secreted by the stimulation of M3 present in parietal cells [104,105,106,107]. In this organ, processes such as cellular proliferation, survival, and tumorigenesis were described upon ACh stimulation; in addition, some authors suggested that ACh acted as a mediator via M3 in gastric cancer cell lines, where M3 and M1 receptors were highly expressed [33,108,109] and M3 antagonists decreased cancer growth in mice gastric cells [110], and losing M3 expression in NMU-induced tumors reduced proliferation rate [107]. The pathways identified in these processes were MAPK, AKT, yes-associated protein (YAP), WNT, and nerve growth factor (NGF), which were determined by the previous activation of M3 and EGFR ([108,111,112,113]. Despite the association of the PI3K/AKT pathway with regulatory processes in mammalian cell survival, relationships with other functions, such as proliferation, growth, and metabolism, were also reported [109]. These functions were associated with M3 activation, along with EGFR stimulation [33]. It was observed that, without or blocking the nervous activity, the tumor growth was diminished [92,108]. Similarly, ACh induced invasion, migration, and epithelial-to-mesenchymal transition (EMT), and such effects were suppressed by a muscarinic antagonist, even in the absence of ACh [92], thus supporting possible crosstalk with another pathway or through the blockage of another receptor. So, stimulation of M3 promoted not only proliferation, but also suppressed apoptosis through EGFR and AKT pathways [111,114].

The communication between the cholinergic system and the EGFR signaling pathway was confirmed by Yu et al. performing in vivo and in vitro studies on gastric cancer cell lines, MKN45 and BGC823. They found that ACh, through the M3, activated EGFR signaling to induce ERK1/2 and AKT phosphorylation. They also found that using the inhibitors U0126 and MK2206 for ERK and AKT, respectively, the proliferation induced by ACh stimulation was reverted. ACh-induced cell proliferation was also inhibited under the effect of AG1478, an EGFR inhibitor, suggesting that ACh might act through M3 to activate EGFR signaling and promote cell proliferation in gastric cancer cells. They also observed that antagonists, such as trihexyphenidyl, M1 antagonist, and selective M2/M4 antagonist AFDX-116, did not affect gastric cell proliferation; however, in vivo studies showed a reduction in tumor size in animals treated with 4-DAMP and darifenacin, both M3 selective antagonists [33]. This supported the established communication between mAChR and EGFR, and the pivotal role of M3 in gastric cancer. Similarly, YAP, which is part of the hippocampus pathway in gastrointestinal tumor cells [115,116], was associated with tumorigenesis by activating stem cells of tissues [117]. Under GPCRs stimulation, especially by Gq/11 and Gi/o phosphorylation, not only other kinases, but also YAP protein was controlled [118,119,120]. For instance, in TMK1 gastric cancer cells, carbachol decreased the phosphorylation of YAP, and YM254890, a Gq/11 specific inhibitor, blocked this effect [107,118]; hence, activation of YAP by blocking its phosphorylation could serve as another possible therapeutic target of nerve-dependent cancers [115,118].

Authors observed that carbachol, a mAChR agonist, upregulated Ngf expression in gastric organoids; the organoids under ACh presence reversed their lack of Ngf expression after their complete isolation; studies in vivo showed that the nerve growth factor (NGF) was overexpressed under ACh in an M3-dependent manner, promoting carcinogenesis in the gastric epithelium and innervation in tumors [107].

In the search for effective biomarkers for diagnosis and prewarning, it was confirmed that *CHRM2* methylation rates increased with progression from normal to gastric precancerous lesions, and then to gastric cancer; however, there was no observable decrease from preoperative to postoperative evaluation [121]. *CHRM2* interacting with the node ADMTS9-AS2 was enriched in the PI3K–Akt signaling pathway, and low ADAMTS9-AS2 expression is linked to the prognosis of patients [122].

#### 2.3.1. Correlation between EGFR Expression and Cholinergic Muscarinic Receptors in Stomach Adenocarcinoma

**Figure 7:**
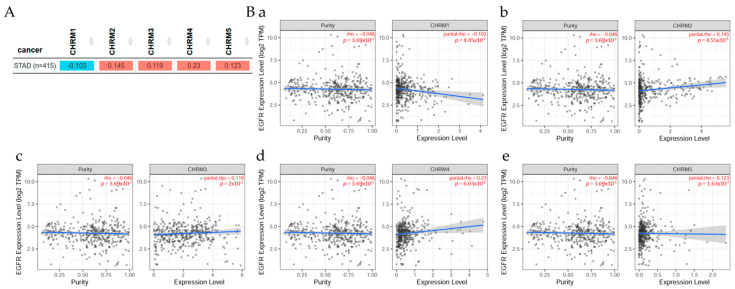
Co-expression pattern of genes across stomach adenocarcinoma (STAD) explored with TIMER2.0. (**A**) The heatmap table, an output from the Gene Corr Module, shows the correlation between *EGFR* expression and cholinergic receptor, muscarinic 1–5 (*CHRM1*–*5*) in STAD with purity adjustment. The red color indicates a statistically significant positive correlation (Spearman’s, *p* < 0.05), blue indicates a statistically significant negative correlation (Spearman’s, *p* < 0.05). Scatter plots show significant correlations between *EGFR* expression and (**B**) (**a**) *CHRM1*, (**b**) *CHRM2*, (**c**) *CHRM3*, (**d**) *CHRM4*, and (**e**) *CHRM5* in STAD (*n* = 415) with tumor purity (left panel) and with the expression level estimated by TIMER2.0 (right panel) [11].

The correlation between *EGFR* expression and cholinergic muscarinic receptors in stomach adenocarcinoma (STAD) is shown in Figure 7. TIMER2.0 analysis tools were used to explore this correlation [11]. (A) There was a significant (*p* < 0.05) positive correlation between *EGFR* and *CHRM2*, *CHRM3, CHRM4*, and *CHRM5* in STAD, but the correlation between *EGFR* and *CHRM1* was significantly (*p* < 0.05) negative. Scatter plots show the correlation between *EGFR* expression and other genes in cancer patients. (B) According to Spearman’s *p* values, results indicated there was a significant (*p* < 0.05) positive correlation between *EGFR* expression and (b) *CHRM2*, (c) *CHRM3*, (d) *CHRM4*, and (e) *CHRM5* expression in STAD, except for *CHRM1*, which was significantly (*p* < 0.05) negative.

#### 2.3.2. Survival Difference in Stomach Adenocarcinoma Adjusted by the Stage Clinical Factor

**Figure 8:**
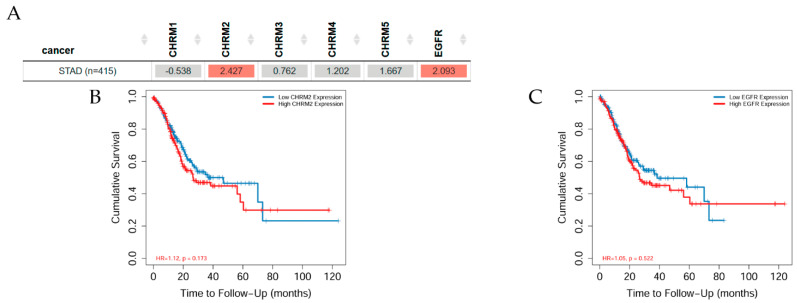
Associations between gene expression and tumor features in TCGA were evaluated with the Timer2.0 Exploration component. (**A**) The heatmap table generated by the Gene Outcome Module shows the survival analysis of cholinergic receptor muscarinic 1–5 (*CHRM1*–*5*) and *EGFR* expression in stomach adenocarcinoma (STAD) adjusted by the clinical-stage factor. The red color indicates a statistically significant increased risk (Z-score, *p* < 0.05) and gray denotes a nonsignificant result. The Kaplan–Meier (KM) curves provide detailed information about the corresponding significant relationship between gene expression and survival of (**B**) *CHRM2* and (**C**) *EGFR* in STAD (*n* = 415) at low and high expression given by cumulative survival and time to follow-up [11].

Overall survival difference among patients stratified by mAChRs expression level in STAD is depicted in Figure 8. There was mostly no significant difference between receptor gene expression and tumor features in stomach cancer adjusted by stage clinical factor; however, *CHRM2* and *EGFR* expressions showed a significant (*p*< 0.05) increased risk in STAD; the Kaplan–Meier (KM) curves indicated that *CHRM2* showed a significant (*p* < 0.01) difference between low and high expression in stage 3 and a significantly (*p* < 0.001) increased risk of survival in stage 4 in STAD (B); *EGFR* expression had a significant increased risk difference between low and high expression in stage 3 (*p*< 0.01) and stage 4 (*p*< 0.001) in STAD.

#### 2.3.3. Association between Gene Expression and Immune Infiltration Level in Stomach Adenocarcinoma

**Figure 9:**
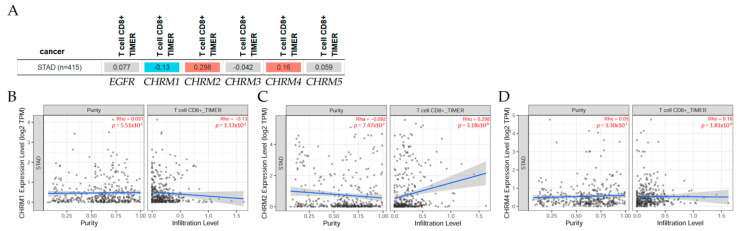
TIMER2.0 Immune component was used to evaluate associations between gene expression and immune infiltrates. (**A**) The heatmap table shows the association between *EFGR*, cholinergic muscarinic receptor expression 1–5 (*CHRM1*–*5*), and immune infiltration level of T cell CD8^+^ in stomach adenocarcinoma (STAD). The red color indicates a statistically significant positive correlation (Spearman’s, *p* < 0.05), blue indicates a statistically significant negative correlation (Spearman’s, *p* < 0.05), and gray denotes a nonsignificant result. Scatter plots show the correlation between (**B**) *CHRM1*, (**C**) *CHRM2*, and (**D**) *CHRM4* expressions and T cell CD8^+^ in STAD (*n* = 415) with tumor purity (left panel) and with the infiltration level of T cell CD8^+^ estimated by TIMER2.0 (right panel) [11].

The correlation of gene expression with immune infiltration levels in diverse cancer types can be seen in Figure 9. The gene module, according to data on TIMER2.0 web analysis tools [11], using Spearman’s *p* values, allowed us to select genes and visualize the correlation of its expression with immune infiltration level in (A) *EGFR* and diverse muscarinic receptor types. (B) Results indicated there was a positive significant (*p* < 0.05) correlation between *CHRM2* gene expression and immune infiltrates, considering the T cell CD8^+^ by TIMER in STAD. (C) There was a positive significant (*p* < 0.05) correlation between *CHRM4* gene expression and immune infiltrates, considering the T cell CD8^+^ by TIMER in STAD, but a negative significant (*p* < 0.05) correlation between *CHRM1* gene expression and immune infiltrates, considering the T cell CD8^+^ by TIMER in STAD.

It can be concluded that there was a positive correlation between *EGFR* expression and *CHRM2*-*5* expression in STAD, except for *CHRM1*, which was negative. Furthermore, the survival differences adjusted by the clinical stage indicated that *CHRM1*–*5* and *EGFR* expression levels were significant (*p* < 0.01) in stage 3, and also significant (*p* < 0.001) in stage 4 in STAD patients. Therefore, targeting cholinergic muscarinic receptors in stomach adenocarcinoma appears to be an attractive therapeutic measurement.

### 2.4. Colorectal Cancer

Colorectal cancer, also known as colorectal adenocarcinoma, is one of the most dangerous cancer-related deaths [123,124], with 1.8 million cases worldwide in 2018 [64], being most common in developed countries, such as Southern and Northern Europe, Australia/New Zealand, and the USA [125]. Different stages can be found in colorectal cancer; according to the American Joint Committee on Cancer (AJCC), a tumor, lymph node, metastasis (TNM) staging system was established; this system was based on the size of the tumor, positive lymph nodes, and the spread of the metastasis [126,127]. Among the risk factor are race, ethnicity, family history, and diet [94,128,129]. In many developed countries, the incidence has decreased due to better tools to prevent, detect early, and effectively treat this cancer, but the mortality rate is still rising in undeveloped countries [130].

Perineural invasion, a type of tumor–nerve interaction that refers to the detection of tumor cells near the nerve, has been identified as a strong and independent prognosis predictor in colorectal cancer; denervation of autonomic and enteric nerves has shown that the presence of these nerves in the gut is accompanied by increased cancer proliferation and growth by releasing neurotransmitters and activating multiple downstream pathways [131].

The cycle of self-renewal of colon cells consists of the migration of specialized epithelium cells, from the crypt to the villus [125]. After around 14 days at the top in the villus, the cells undergo apoptosis for eventual elimination in the feces [132]. This renewal process is highly regulated and is relevant for colorectal cancer stem cell behavior; some of the pathway elements involved in this self-renewal process are Wnt, Notch, EGFR/MAPK, NF-kB, and AKT/mTOR, among others [133]. Regarding EGFR, after its activation, it undergoes dimerization and phosphorylation, which activates MAPK, AKT, and JAK-STAT downstream [134]. EGFR inhibitors cause apoptosis and stop the proliferation process [128]. However, it was demonstrated that mAChRs were present in a great number of human colon cancer cell lines [129] and the ChAT enzyme was also observed in these cells being synthesized and released to stimulate proliferation in an autocrine/paracrine manner [135]. M3 is the main receptor in most colon cancer cells and has been associated with colorectal cancer progression [113,136], when these receptors are overexpressed; they can establish communication with some growth-regulatory pathways [137,138] and induce matrix metalloproteinase-1 (MMP1), involved in processes such as metastasis [139].

The mAChRs have two endogenous ligands, the ACh and the conjugated secondary bile acids [140]. Once the M3 is activated, both pathways, the EGFR-dependent and -independent, are activated with effects on colon cancer progression [136]. Like in other cancer cells, the activation of M3 in colon cancer cells induces transactivation of EGFR [141]. The activation of EGFR by ACh and bile acids in colon cancer cells was studied, confirming the activation of M3 in proliferation via MAPK (ERK1/2) [141,142]. Unconjugated secondary bile acids induced M3 and EGFR expression in normal human colonic epithelial cells, therefore establishing M3 as essential in colon cancer initiation [143]. Studies on cell proliferation showed that muscarinic agonists, such as carbamylcholine, exerted a mobilization of intracellular calcium in colon cancer cells, serving as a second messenger downstream [54]. In an in vivo study using M3 knockdown mice, a well-known carcinogenic drug was used to see if proliferation and neoplasia were directly related to muscarinic presence; animals without M3 expression and under the effects of azoxymethane did not present multiple adenocarcinomas and, for those animals that presented tumors, their sizes were 60% smaller than those wild-type mice under the presence of the same carcinogen [113], therefore supporting the role of M3 in the initiation and proliferation of colon cancer.

Irinotecan is a prodrug derived from an alkaloid plant and it is used in several tumors, including ovarian, rectal, and cervical [144]. Unfortunately, using these drugs has severe side effects on patients [145,146,147]. In these cases, using anticholinergic drugs, such as scopolamine and butylbromide, has improved these effects [148,149].

In vitro and in vivo research with M3 antagonists demonstrated that colon cancer cell proliferation and growth were reduced [80,96]; a study performed in Denmark with 72,917 patients found an association between exposure to antimuscarinics and the risk of lung and colon cancer [32,80,150,151]. Other authors also demonstrated the same relationship between antimuscarinic drugs and the incidence of lung and colon cancer [96].

Colon malignancies overexpress the *CHRM3* gene and protein, and post-mAChR M3 signaling promotes cell proliferation; the interplay between EGFR/ERK and protein kinase C (PKC)/p38 mitogen-activated protein (MAP) kinase signaling pathways is complex after mAChR M3 signaling; then, the formation of an invasive and metastatic phenotype requires these signaling interactions to increase the cellular release of MMP1; hence, targeting mAChR M3, post-mAChR M3 signaling, or MMP1 to prevent or reverse colon cancer invasiveness offers therapeutic potential [136]. Bethanechol treatment increased the expression of *CHRM3*, EGFR, and post-EGFR signaling molecules Myc and cyclin D1 in colon cancer; it also increased the thickness of normal colonic mucosa and the expression of selected MMP genes, such as *MMP7*, *MMP10*, and *MMP13*, indicating that mAChRs play a key role in colon neoplasia and pointing to post-receptor signaling molecules as potential therapeutic targets [152].

#### 2.4.1. Correlation between EGFR Expression and Cholinergic Muscarinic Receptor in Colon Adenocarcinoma

**Figure 10:**
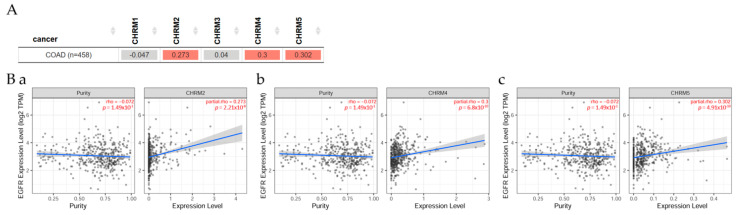
Co-expression pattern of genes across colon adenocarcinoma (COAD) evaluated by Timer2.0 Exploration components. (**A**) The heatmap table, an output from the Gene Corr Module, shows the correlation between *EGFR* expression and cholinergic receptor muscarinic 1–5 (*CHRM1*–*5*) in COAD with purity adjustment. The red color indicates a statistically significant positive correlation (Spearman’s, *p* < 0.05) and gray denotes a nonsignificant result. Scatter plots show the correlation between *EGFR* expression and (**B**) (**a**) *CHRM2*, (**b**) *CHRM4*, and (**c**) *CHRM5* in COAD (*n* = 458) with tumor purity (left panel) and with the expression level estimated by TIMER2.0 (right panel) [11].

Gene correlation between *EGFR* expression and cholinergic muscarinic receptor in colon adenocarcinoma (COAD) can be seen in Figure 10. Results indicated there was a significant difference (*p* < 0.05) between EGFR and *CHRM2*, *4*, and *5* in COAD patients. However, overall survival among patients stratified by mAChRs expression level indicated there was no significant difference between receptor gene expression and tumor features in COAD adjusted by stage clinical factor.

#### 2.4.2. Association between Gene Expression and Immune Infiltration Level in Colon Adenocarcinoma

**Figure 11:**
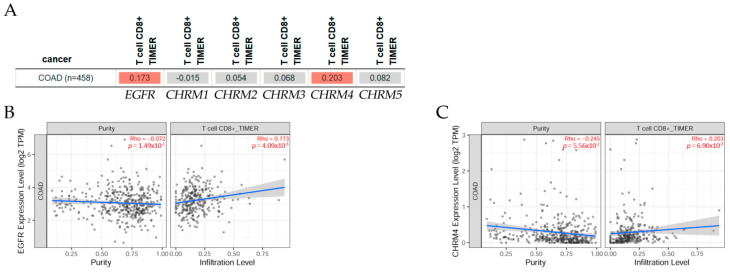
TIMER2.0 Immune component was used to evaluate associations between immune infiltrates and gene expression. (**A**) Heatmap table shows the association between *EFGR*, cholinergic muscarinic receptor expression 1–5 (*CHRM1*–*5*), and immune infiltration level of T cell CD8^+^ in colon adenocarcinoma (STAD). The red color indicates a statistically significant positive correlation (Spearman’s, *p* < 0.05) and gray denotes a nonsignificant result. Scatter plots show the correlation between (**B**) *EGFR* and (**C**) *CHRM4* expressions in COAD (*n* = 458) with tumor purity (left panel) and with the infiltration level of T cell CD8^+^ estimated by TIMER2.0 (right panel) [11].

The gene module according to data on TIMER2.0 web analysis tools [11] shows the correlation of gene expression with immune infiltration levels in diverse cancer types (Figure 11); using Spearman’s *p* values allowed us to select genes and visualize the correlation of their expression with immune infiltration level in (A) *EGFR* and diverse muscarinic receptor types. Results indicated there was a positive significant (*p* < 0.05) correlation between (B) EGFR, (C) *CHRM4* gene expression, and immune infiltrates, considering the T cell CD8^+^ by TIMER in COAD, but not significant between *CHRM1*, *CHRM2*, *CHRM3*, *CHRM5*, and T cell CD8^+^ in COAD.

In summary, there was a positive correlation between *EGFR* and *CHRM2*, *4*, and *5* expression levels in COAD patients. The survival differences adjusted by the clinical stage indicated that *CHRM1*–*5* and *EGFR* expression levels were significantly (*p* < 0.05) higher in stage 3 and also significantly (*p* < 0.001) higher in stage 4 than the other stages in COAD patients. It is important to emphasize that the cholinergic muscarinic receptors appear to be a late marker for the disease in comparison with other markers.

### 2.5. Liver Cancer

Liver cancer has been recognized as a heterogeneous disease with several histological characteristics [153]: it is the fourth most common death-related cancer worldwide [64], its mortality and incidence are among those tumors with a high increase [33,34], it has a low prognosis [153], and the worldwide incidence rate is higher in males than in females [154]. The assistance of new imaging techniques, such as magnetic resonance or computerized tomography has allowed the detection of pathological features with more sensitivity [155,156]. Generally, liver cancer comprises a group of tumors from the most common ones, such as hepatocellular carcinoma (HCC) or intrahepatic cholangiocarcinoma (iCCA), to other more rare types, such as hepatoblastoma and fibrolamellar carcinoma, or mixed types, such as the mixed hepatocellular cholangiocarcinoma (HCC-CCA), fibrolamellar HCC (FLC), or the pediatric neoplasm hepatoblastoma [157,158,159]. A correlation between genetic alterations and signaling pathways that lead to tumor progression was observed in these cancer cells [150,151,160].

A classification was suggested integrating both the morphological phenotypes and molecular alterations [157]. While HCC, one of the most recurrent primary malignant liver tumors, was classified in proliferative and non-proliferative tumors [161,162], considering its molecular and histopathological features with subclassifications involving grade of differentiation or oncogenic pathways, such as RAS/MAPK and PI3K/AKT in the proliferative type and JAK/STAT and Wnt/b catenin in the non-proliferative one [157]. HCC is usually associated with chronic hepatitis B (HBV) or C (HCV) virus infection [163]. Among the risk factors reported for liver cancer are alcohol intake or metabolic syndrome, obesity, and diabetes [161,162,163]. Around 18 million people abuse alcohol in the US, increasing the risk for HCC; this dramatically increases if the alcohol intake comes with chronic hepatitis C [158,163]. Regarding the mortality and prevalence, IARC indicated that mortality was high in Asia, with 566269 cases, followed by Europe and Africa; similarly, the 5-year prevalence in males and females was led by Asia, with Europe and Africa in third place [154].

Neurotransmitter-related genes associated with cell proliferation and survival in HCC were reported over 10 years ago [159]. Despite the full characterization regarding the innervation of the sympathetic and parasympathetic system, it has not been achieved in HCC yet; however, mAChRs were positively reported in this specific cancer and hepatoma cells, and it was reported that M1 and M3 receptor expression was associated with tumor progression and poor prognosis in patients [164]. In human liver tumor samples, low expression of AChE was detected when compared with normal tissue samples [38]. Considering this enzyme is fundamental in the degradation of ACh, its presence showed a reduction in cell proliferation in hepatoma cells [165,166], suggesting a tumor growth suppressor role of this enzyme [159,167], perhaps by diminishing ACh. The absence or low expression of this enzyme has also been related to the aggressiveness of HCC in both in vitro and in vivo studies [168]. This neurotransmitter was widely proposed as a regulator of cell proliferation, differentiation, and cell–cell adhesion [169]. It was observed that vesicular acetylcholine transported (VAChT) was also expressed in HCC tissue samples, resulting in malignant characteristics, such as metastasis, vascular invasion, and recurrence [164].

The expression of AChE was downregulated in mainly all HCC human tissue samples analyzed, and a high risk of cancer recurrence and poor prognosis was observed in patients with low expression of the enzyme [168]. A study showed EGFR was overexpressed in about 68% of human HCC and was correlated with aggressive tumors, metastasis, and poor survival [170,171,172]. A polymorphism in the EGF gene was associated with a high risk for HCC in cirrhotic patients [173,174], and EGF was upregulated in those patients [175,176]. Data on possible communication between EGFR and the cholinergic ligand indicated that overexpression of AChE was detected in HCC cells blocking the activation of MAPK and PI3K/AKT signaling pathway by decreasing the phosphorylation of ERK and AKT; this overexpression also produced an enhancement of the pharmacological effect of drug-induced apoptosis [168].

In another in vitro study, the stimulation of M1 induced cell migration, invasion, and EMT in HepG2 and SMMC-7721 by the PI3K/AKT pathway; such effect was counteracted by a muscarinic antagonist or shRNA [177]. A link with an inflammatory and tumor-promoting pathway in HCC cells was suggested and a communication possible is due to the induction of amphiregulin by TNF-alpha, allowing the transactivation of EGFR [178]. Within this context, some EGFR inhibitors emerged as an alternate treatment for HCC: Gefitinib, Erlotinib, and Lapatinib showed good results in animal models [179,180,181]. In a clinical setup, either Gefitinib or Lapatinib showed no efficiency [182,183], while, with Erlotinib, a moderate effect was observed [184,185,186]. Likewise, when a combination of Erlotinib and Bevacizumab (a selective VEGF inhibitor) was used, a moderate effect was observed as well [187,188]. Since the sole inhibition of EGFR is not efficient enough, another HCC therapeutic alternative must be explored.

A study reported that ACh stimulated cell migration and invasion in HCC with inhibition of the apoptotic process [189], fundamental in cancer metastasis, and such actions were associated with androgen receptors (AR) [190]. Perhaps this relation could be considered in the explanation of a higher incidence of HCC in males than in females [191,192]. Specifically, in SNU-449 cells, ACh induced AR protein expression and it also activated the same receptor, thus controlling migration, invasion, and apoptosis [190]. The AR could also be activated by other ligands, such as IL-6 and HER2/Neu signaling-related molecules [193]. Liver hepatocellular carcinoma (LIHC) is a primary malignancy with no viable treatment for advanced patients; nevertheless, *CHRM3* and five other potential prognostic markers have been identified by investigating the relationship between genes and patient survival and could be used as therapeutic targets [184].

#### 2.5.1. Gene Correlation between EGFR Expression and Genes Associated with mAChRs in Liver Hepatocellular Carcinoma

**Figure 12:**
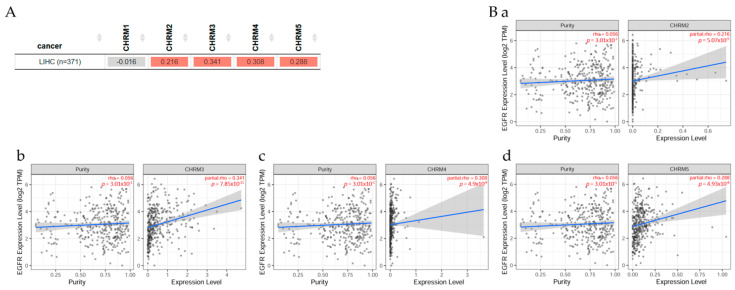
Co-expression pattern of genes in liver hepatocellular carcinoma (LIHC) explored with TIMER2.0. (**A**) The heatmap table, an output of the Gene Corr Module, shows the correlation between *EGFR* expression and cholinergic receptor muscarinic 1–5 (*CHRM1*–*5*) in LIHC with purity adjustment. The red color indicates a statistically significant positive correlation (Spearman’s, *p* < 0.05) and gray denotes a nonsignificant result. Scatter plots show the significant correlations between *EGFR* expression and (**B**) (**a**) *CHRM2*, (**b**) *CHRM3*, (**c**) *CHRM4*, and (**d**) *CHRM5* in LIHC (*n* = 371) with tumor purity (left panel) and with the expression level estimated by TIMER2.0 (right panel) [11].

Gene correlation between *EGFR* expression and genes associated with mAChRs in LIHC patients (Figure 12) indicated there was a positive correlation (*p* < 0.05) between *EGFR* and *CHRM2*, *CHRM3, CHRM4,* and *CHRM5* in liver cancer. Scatter plots show the significant (Spearman’s, *p* < 0.05) positive correlation between *EGFR* and (a) *CHRM2*, (b) *CHRM3*, (c) *CHRM4*, and (d) *CHRM5* gene expression in LIHC.

#### 2.5.2. Association between Gene Expression and Immune Infiltration Level in Liver Hepatocellular Carcinoma

**Figure 13:**
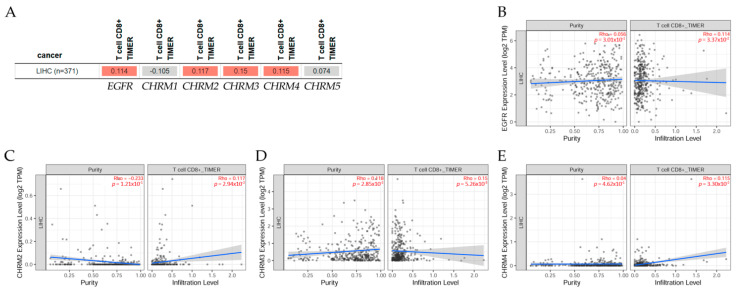
Associations between immune infiltrate and gene expression evaluated with TIMER2.0 Immune component. (**A**) The heatmap table shows the association between *EGFR*, cholinergic muscarinic receptor expression 1–5 (*CHRM1*–*5*), and immune infiltration level of T cell CD8^+^ in liver hepatocellular carcinoma (LIHC). The red color indicates a statistically significant positive correlation (Spearman’s, *p* < 0.05) and gray denotes a nonsignificant result. Scatter plots show the correlation between (**B**) *EGFR*, (**C**) *CHRM2*, (**D**) *CHRM3*, (**E**) *CHRM4* expression, and T cell CD8^+^ in LIHC (*n* = 371) with tumor purity (left panel) and with the infiltration level of T cell CD8^+^ estimated by TIMER2.0 (right panel) [11].

The gene module according to data on TIMER2.0 web analysis tools [11] shows the correlation of gene expression with immune infiltration levels in liver hepatocellular carcinoma (LIHC) in Figure 13. Using Spearman’s *p* values allowed us to select genes and visualize the correlation of their expression with immune infiltration levels in (A) EGFR and diverse muscarinic receptor types. (B) Results indicated there was a positive significant (*p* < 0.05) correlation between (B) *EGFR*, (C) *CHRM2*, (D) *CHRM3*, and (E) *CHRM4* gene expression and immune infiltrates, considering the T cell CD8^+^ by TIMER in LIHC, but it was not significant between *CHRM1*, *CHRM5*, and the infiltrate estimation value.

It can be concluded there was a positive correlation between *EGFR* and *CHRM2-5* in LIHC. The survival differences adjusted by the clinical stage indicated that *CHRM1*–*5* and *EGFR* expression levels were significant (*p* < 0.001) in stage 3 and also significant (*p* < 0.01) in stage 4 in LICH. Therefore, targeting cholinergic muscarinic receptors appears to be an attractive therapeutic alternative for liver cancer in later stages.

### 2.6. Prostate Cancer

A deregulated expression of miRNA and an upregulated expression of mAChRs were observed in human prostate cancer tissue [185,194]. In addition, in vitro studies confirmed this abnormal expression of microRNA (miRNA) in prostate cancer cells, reporting changes in adhesion, migration, invasion, and cell cycle distribution [185]. Particularly, miR-30e was highlighted as a possible candidate for future pharmaceutical targets, since it inhibits MAPK signaling pathway by the modulation of the M3 receptor, which was reported to be a growth-suppressive actor in prostate cancer cells [185,186].

In prostate cancer, *CHRM3* was demonstrated to promote growth; carbachol, a cholinergic agonist, showed a proliferative effect due to its agonistic activity on *CHRM3*; in early-stage human prostate cancer tissues, *CHRM1* expression was high and appeared to play a role in prostate cancer proliferation and growth; but, using selective *CHRM1* antagonists, such as pirenzepine and dicyclomine, showed significant inhibition of prostate cancer cell proliferation [186], suggesting that antagonism of *CHRM1* could be a viable therapeutic target for prostate cancer [195].

#### 2.6.1. Correlation between EGFR Expression and Cholinergic Muscarinic Receptor in Prostate Adenocarcinoma

**Figure 14:**
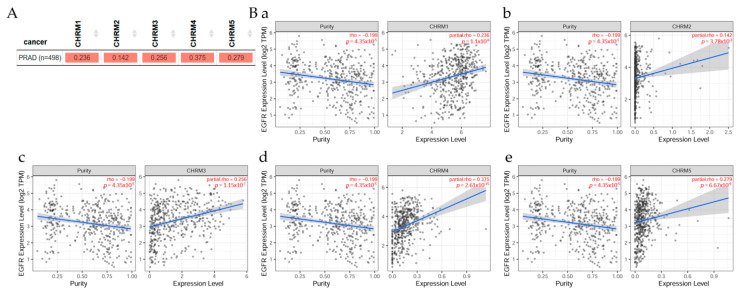
Co-expression pattern of genes across prostate adenocarcinoma (PRAD) explored with TIMER2.0. (**A**) The heatmap table, an output from the Gene Corr Module, shows the correlation between *EGFR* expression and cholinergic receptor, muscarinic 1–5 (*CHRM1*–*5*) in PRAD with purity adjustment. The red color indicates a statistically significant positive correlation (Spearman’s, *p* < 0.05). Scatter plots show significant correlations between *EGFR* expression and (**B**) (**a**) *CHRM1*, (**b**) *CHRM2*, (**c**) *CHRM3*, (**d**) *CHRM4*, and (**e**) *CHRM5* in PRAD (*n* = 498) with tumor purity (left panel) and with the expression level estimated by TIMER2.0 (right panel) [11].

Gene correlation between *EGFR* expression and cholinergic muscarinic receptor in prostate adenocarcinoma (PRAD) can be seen in Figure 14; TIMER2.0 analysis tools were used to explore this correlation [11]. (A) There was a significant (*p* < 0.05) positive correlation between *EGFR* expression and *CHRM1*, *CHRM2*, *CHRM3*, *CHRM4*, and *CHRM5* gene expression in PRAD. Scatter plots show the correlation between *EGFR* expression and other genes in cancer patients. (B) According to Spearman’s *p* values, results indicated there was a significant (*p* < 0.05) positive correlation between EGFR expression and (a) *CHRM1*, (b) *CHRM2*, (c) *CHRM3*, (d) *CHRM4*, and (e) *CHRM5* expression in PRAD.

#### 2.6.2. Association between Gene Expression and Immune Infiltration Level in Prostate Adenocarcinoma

**Figure 15:**
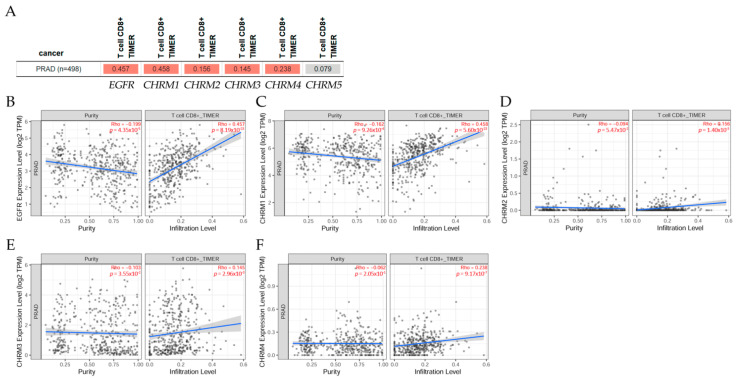
Associations between immune infiltrate and gene expression were evaluated with the TIMER2.0 Immune component. (**A**) Heatmap table shows the association between *EGFR*, cholinergic muscarinic receptor expression 1–5 (*CHRM1*–*5*), and immune infiltration level of T cell CD8^+^ in prostate adenocarcinoma (PRAD). The red color indicates a statistically significant positive correlation (Spearman’s, *p* < 0.05) and gray denotes a nonsignificant result. Scatter plots show the correlation between (**B**) *EGFR*, (**C**) *CHRM1*, (**D**) *CHRM2*, (**E**) *CHRM3*, (**F**) *CHRM4* expression and T cell CD8^+^ in PRAD (*n* = 498) with tumor purity (left panel) and with the infiltration level of T cell CD8^+^ estimated by TIMER2.0 (right panel) [11].

The gene module according to data on TIMER2.0 web analysis tools [11] correlates gene expression with immune infiltration levels in prostate adenocarcinoma (PRAD) in Figure 15. Using Spearman’s *p* values allowed us to select genes and visualize the correlation of their expression with immune infiltration levels in (A) *EGFR* and diverse muscarinic receptor types. (B) Results indicated there was a positive significant (*p* < 0.05) correlation between (B) EGFR, (C) *CHRM1*, (D) *CHRM2*, (E) *CHRM3*, and (F) *CHRM4* gene expression and immune infiltrates, considering the T cell CD8^+^ by TIMER in PRAD, but it was not significant between *CHRM5* and T cell CD8^+^.

It can be concluded there was a positive correlation between *EGFR* expression and *CHRM1*–*5* gene expression in PRAD. Results indicated that *CHRM1*–*5* and *EGFR* expression levels were nonsignificant in any of the clinical stage in PRAD. Thus, targeting cholinergic muscarinic receptors could not be conducted according to the clinical stages. However, T cell CD8^+^ would be a good marker for this type of cancer.

### 2.7. Glioblastoma Multiforme

Glioblastoma multiforme (GBM) is one of the most aggressive and deadly brain tumors in patients; its invasive behavior increases under the influence of Ach [196]. There was a correlation between M3 overexpression and a decrease in patient survival, and with an increase in the MMP-9 activity in the presence of muscarinic agonists [197]. It has been reported that EGFR and Notch signaling pathways are the most unbalanced in GBM cell lines [198], the former because of its hyper-activation in several tumor cells [199,200], and the latter due to its effects on tumor progression [201,202].

On the hand, authors studied these interactions in human glioblastoma cell lines (U251MG and U87MG) and reported that, under arecaidine propargyl ester (APE), a muscarinic agonist, the M2 receptors were activated, which induced upregulation of miRNA 34a-5p expression, and that would eventually downregulate the expression of both Notch-1 and EGFR [203]. These results also gave new inputs for a new possible therapeutic use of microRNA technology.

GBM is characterized by heterogeneous cell populations that have the potential of mAChR M2 agonist to overcome drug resistance in two glioblastoma stem cell (GSC) lines, which was was revealed by downregulation of the expression of ATP-binding cassette (ABC) drug efflux pumps; hence, mAChR M2 agonists combined with low dosages of traditional chemotherapy may provide a new potential pharmacological method to impair GSC drug resistance in GBM therapy [204].

TIMER2.0 web tools [11] indicated that gene correlation between *EGFR* expression and genes associated with mAChRs (*CHRM1*–*5*) was not significant in GBM patients; the Gene Outcome module indicated that survival of these genes was not significant either, and the Immune Module showed that immune infiltration level in GBM was not significant. Results also indicated that *CHRM1*–*5* and *EGFR* expression levels were nonsignificant in any of the clinical stages in GBM, avoiding a therapeutical solution for patients with this fatal disease.

The mAChRs were expressed in tumor cells, and, among them, the M3 receptor appeared to be involved in tumor progression by muscarinic activation that induced cell proliferation [38]. Thus, blocking the M3 receptor inhibited in vivo and in vitro proliferation. Furthermore, modulation of cell growth was observed by receptor agonists and antagonists. It is important to mention that abnormal activation of EGFR family kinases, including ErbB1 (HER1), ErbB2 (HER2), ErbB3 (HER3), and ErbB4 (HER4), resulted in excessive cell proliferation and angiogenesis. On the other hand, the composition and abundance of immune cells in the tumor microenvironment had a significant impact on tumor progression and immunotherapy efficacy. ACh and cholinergic-related components were also demonstrated in several cancers, among them, the human lung cancers, inducing adhesion, migration, and invasion via a cholinergic autocrine loop. In addition, squamous cell carcinoma of the lung tumors expressed ChAT, suggesting a role of the cholinergic system in the progression of lung cancers. Overexpression of M3 was observed in NSCLC patients with poor survival rates, but it was reported that ACh also served as a paracrine and autocrine growth factor for bronchial epithelial cells, SCLCs, SCC-Ls, and lung adenocarcinomas. Crosstalk between the M3 receptor and EGFR was suggested for cancer, for instance, in lung enhancing processes, such as proliferation, migration, and invasion due to ACh activity. Studies on the effects of different muscarinic agonists on lung cancer indicate that the activation of the EGFR/PI3K/AKT pathway is due to M3 activation [17,79,81,82,86].

A correlation between EGFR expression and genes associated with cholinergic muscarinic receptors was observed in lung adenocarcinoma and lung squamous cell carcinoma. There was a positive correlation between EGFR expression and genes associated with mAChRs in lung adenocarcinoma (LUAD) and lung squamous cell carcinoma (LUSC) patients. Association between gene expression and immune infiltration levels in EGFR and diverse muscarinic receptor types were observed in lung adenocarcinoma and lung squamous cell carcinoma among several types of cancer.

**Scheme 1:**
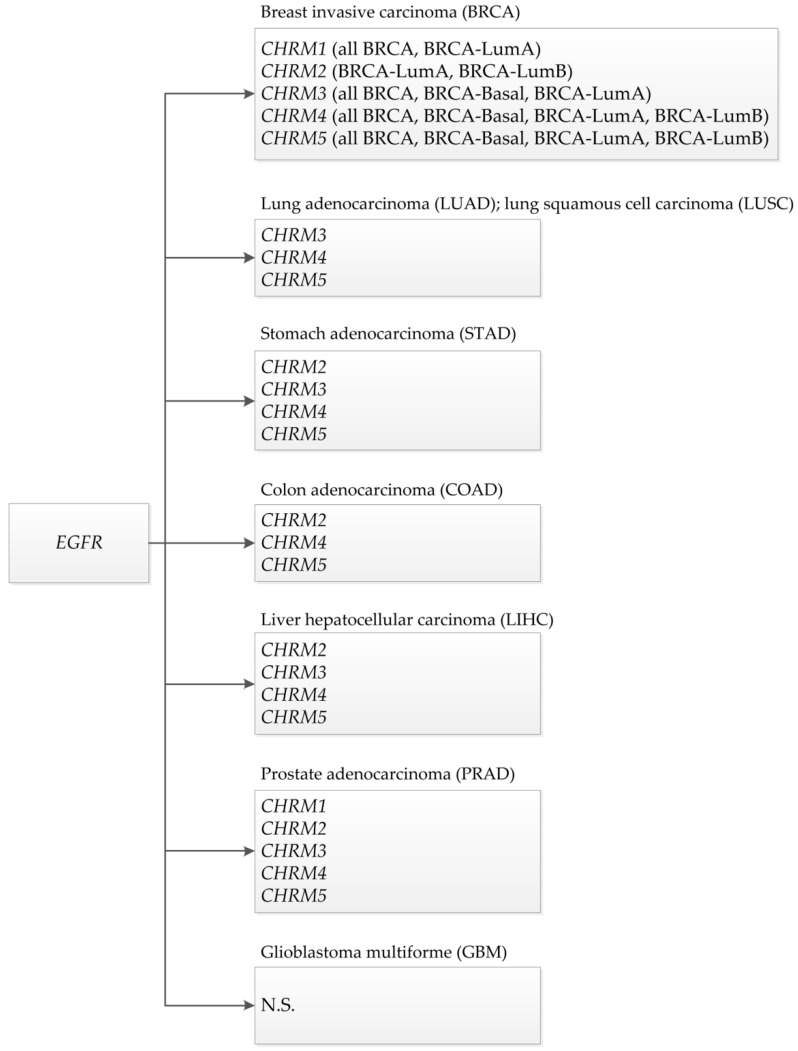
Correlation between *EGFR* and cholinergic muscarinic receptors 1–5 in breast, lung, stomach, colon, liver, prostate, and glioblastoma human cancers.

This review showed that muscarinic receptors, such as *CHRM2*, *3*, *4*, and *5*, were altered in breast, stomach, lung, colon, liver, and prostate cancers. *CHRM4* and *5* receptors were altered in breast (BRCA-LumA, BRCA-LumB, and BRCA-Basal), stomach, lung (LUAD and LUSC), colon, liver, and prostate cancers; *CHRM3* receptor was altered in breast (BRCA-LumA and BRCA-Basal), stomach, lung (LUAD and LUSC), liver, and prostate cancer; *CHRM2* was altered in breast (BRCA-LumA and BRCA-LumB), stomach, colon, liver, and prostate cancer; and *CHRM1* receptor was altered in breast (BRCA-LumA), stomach, and prostate cancer (Scheme 1). *CHRM1*–*5* showed no significant differences in breast (BRCA-Her2) and glioblastoma. These studies also showed that the overall survival among patients was not significantly different between receptor gene expression and tumor features in breast invasive carcinomas or colon adenocarcinomas. However, *CHRM3* gene expression showed an increased risk in breast cancer (BRCA-LumA); M4 receptor gene expression showed an increased risk in BRCA-Basal, but a decreased risk in BRCA-Her2 adjusted by stage clinical factor.

## 3. Conclusions

Cancer is a multistep disease with complex processes and with particular cellular behaviors. Thus, the emergence of new alternatives for cancer treatment is imperative to tackle the disadvantages of classical pharmacological interventions. There is an established pharmacological protocol for tumors, and patients with similar molecular features at the same stage can respond differently to pharmacological approaches when they have a different prognosis [205]. Certain cancers overexpress certain receptors and its mediation is normally related to other effectors, such as EGFRs and *CHRM1*–*5*. Therefore, a synergetic effect after stimulating those receptors should be important to consider.

Then, the ability of muscarinic agonists to stimulate growth and M3 receptor antagonists to inhibit tumor growth has been demonstrated for breast, melanoma, lung, gastric, colon, pancreatic, ovarian, prostate, and brain cancer. Cholinergic machinery is present in a wide variety of cancers, whereas mAChRs have been exhibited to be organ-specific and the outcome will depend on the environment of the specific cancer cell type. Several data confirm the communication of the mAChR activation and other signaling pathways associated with tumor progression, providing a wide overview of their contribution to different cancer formation and development for new antitumor targets.

In conclusion, there is a correlation between epidermal growth factor receptors and cholinergic muscarinic receptors, survival clinical differences adjusted by stage factor, and an association between gene expression and immune infiltration level in breast, lung, stomach, colon, liver, prostate, and glioblastoma human cancers. Thus, targeting mAChRs appears to be an attractive therapeutic alternative due to their multiple ligands/second messengers and complex signaling pathways involved. Finally, new pharmacotherapies represent good alternatives to clinical issues, such as the resistance to common therapies and the aggressiveness of some tumors; it is crucial to identify new therapeutic targets to improve the survival of patients considering that carcinogenesis is an interconnected process, involving the hormonal, neuronal, and immune systems.

## Data Availability

TIMER2.0 is freely available at http://timer.cistrome.org (accessed on 3 September 2021).
